# Early-onset intrahepatic cholestasis of pregnancy resulting from a genetic mutation: A case report

**DOI:** 10.1016/j.crwh.2025.e00714

**Published:** 2025-05-13

**Authors:** Alyssa Gonzalez, Courtney Fant, Ashten Waks, Thao Le, Jonathan G. Steller

**Affiliations:** a Department of Obstetrics and Gynecology, University of California Irvine Medical Center, 3800 W Chapman Ave, Ste 3400, Orange, CA 92868, USA; b Division of Maternal-Fetal Medicine, Department of Obstetrics and Gynecology, University of California Irvine Medical Center, 3800 W Chapman Ave, Suite 3400, Orange, CA 92868, USA; c University of California Irvine Medical School, 1001 Health Sciences Rd, Irvine, CA 92697, USA

**Keywords:** Benign recurrent intrahepatic cholestasis (BRIC), Intrahepatic cholestasis of pregnancy (ICP), Jaundice in pregnancy

## Abstract

Intrahepatic cholestasis of pregnancy (ICP) is a pregnancy-specific condition characterized by pruritus and elevated bile acids. It typically manifests in the third trimester of pregnancy and has been associated with hormonal and genetic factors. Early-onset ICP poses unique diagnostic challenges and may contribute to increased risks of adverse maternal and fetal outcomes. We present a case of severe ICP identified in the early second trimester and later attributed to a rare gene variant.

A 24-year-old patient (G3P0202) was admitted at 17 weeks of gestation with pruritis, abdominal pain, and jaundice. Laboratory studies were notable for elevated total and direct bilirubin as well as elevated bile acids. The patient's medical history included early-onset ICP accompanied by jaundice in all previous pregnancies. A cholestasis gene panel revealed an autosomal recessive variant in the ABCB11 gene, which encodes a bile salt export pump and is associated with benign recurrent intrahepatic cholestasis (BRIC). Throughout the duration of her pregnancy, the patient was co-managed with the hepatobiliary service, and her symptoms were adequately controlled with ursodeoxycholic acid, rifampin, and hydroxyzine. She labored spontaneously at 34 weeks of gestation and delivered a healthy infant.

This case underscores the importance of genetic evaluation in the assessment of atypical ICP, particularly in early-onset, recurrent, or treatment-refractory cases. It also highlights the need for multidisciplinary management of complex cases with obstetricians, hepatologists, and genetic counselors.

## Highlights

• The importance of genetic evaluation for women presenting with atypical intrahepatic cholestasis of pregnancy.
• Intrahepatic cholestasis of pregnancy can be a cause of jaundice in pregnancy.
• Atypical intrahepatic cholestasis in pregnancy includes early-onset, recurrent, and treatment-refractory cases.

## 1 Introduction

Intrahepatic cholestasis of pregnancy (ICP) is a disorder that usually manifests in the third trimester of pregnancy with severe pruritis and elevated serum bile acids. The incidence is estimated to range from 0.3 % to 15 % in different populations [1]. ICP is of concern because it has been associated with adverse perinatal outcomes, including increased rates of spontaneous and iatrogenic preterm delivery, meconium-stained amniotic fluid, fetal arrhythmia, and stillbirth [2]. While the pathophysiology of ICP is not well understood, it is thought to be associated with hormonal and genetic factors. Symptoms and bile acids often improve spontaneously after delivery, though patients remain at significant risk of recurrence in subsequent pregnancies. We present a case of severe, atypical ICP identified in the early second trimester and later attributed to a rare gene variant.

## 2 Case Presentation

A 24-year-old patient (G3P0202) was admitted at 17 weeks of gestation with pruritis, abdominal pain, and jaundice. Her medical history was significant for early-onset ICP accompanied by jaundice in both of her previous pregnancies. She was diagnosed at 11 weeks of gestation in her first pregnancy, necessitating delivery at 36 weeks. She had inconsistent prenatal care during her second pregnancy and was delivered via cesarean section at 32 weeks of gestation for preterm labor and malpresentation. She denied any history of hepatobiliary or pancreatic conditions outside of pregnancy in herself or close family members, but recalled that a cousin had similar symptoms in her pregnancies.

Prior to admission, the patient's obstetrician diagnosed her with presumed ICP based on serum bile acids of 234 umol/L and had begun treating her with ursodeoxycholic acid 300 mg every eight hours in addition to hydroxyzine as needed for itching. The initial laboratory studies on the present admission were otherwise notable for total bilirubin elevated to 12.1 mg/dL, direct bilirubin elevated to 6.1 mg/dL, and normal liver function tests. Though ICP remained in the differential diagnosis, the onset early in the second trimester and accompanying severe hyperbilirubinemia led to the consideration of other extrahepatic and intrahepatic causes of cholestasis. Hepatology and genetics consultations were requested.

Hepatitis serologies, autoimmune markers, and a metabolic workup were all unremarkable. The patient was initially coagulopathic with an INR of 2.31; however, this normalized after administration of vitamin K and was later attributed to cholestasis-related malabsorption. Right upper quadrant ultrasound and magnetic resonance cholangiopancreatography (MRCP) demonstrated gallstones, but were without evidence of biliary duct dilation, choledocholithiasis, or acute cholecystitis. The patient was continued on ursodeoxycholic acid during this time and was also started on rifampin 150 mg twice daily by the hepatology team for severe refractory itching. After demonstrating stability from a laboratory and symptom standpoint, she was discharged home to follow-up outpatient care with maternal-fetal medicine, genetics, and hepatology.

Outpatient, maternal serum was sent for a cholestasis gene panel. It revealed a pathogenic variant in a single ABCB11 gene. Variants in this gene have been associated with autosomal recessive benign recurrent intrahepatic cholestasis (BRIC) and autosomal recessive progressive familial intrahepatic cholestasis 2 (PFIC-2). Because PFIC-2 tends to be associated with severe, progressive disease and this patient's course remitted between pregnancies, a diagnosis of BRIC was favored. Moreover, because these syndromes are both autosomal recessive and the patient had a single pathogenic variant, a multifactorial inheritance pattern was suspected.

The patient was readmitted at 33 weeks of gestation after self-discontinuing her prescribed medications and presenting again with severe symptoms. At the time of admission, her bile acids were found to be 223 umol/L, though her total bilirubin and INR remained within normal limits (1.1 mg/dL and 0.90, respectively). Based on the extent of her bile acid elevation and concern for fetal morbidity, the decision was made for delivery at 35 weeks. However, she then experienced spontaneous preterm labor at 34 weeks of gestation and delivered a healthy male infant, after which she was lost to follow-up.

## 3 Discussion

Because ICP increases the risk of preterm delivery, fetal distress, and stillbirth, timely diagnosis is imperative for guiding intervention and timing of delivery. As patients with early-onset ICP—those diagnosed before 30 weeks of gestation—have higher rates of these complications, diagnosis in the second and early third trimesters is even more critical [3]. Although patients with ICP usually experience pruritis without a rash, other hepatobiliary diseases and pregnancy-related causes of pruritis, including pemphigoid gestationis, polymorphic eruption of pregnancy, atopic eruption, and pustular psoriasis, must be considered [4]. Thus, the evaluation of pruritus in pregnancy should involve a systematic approach with a detailed history, physical examination, and targeted laboratory investigations to distinguish between the aforementioned causes of pruritis in pregnancy. If ICP is suspected, evaluation should include serum bile acids and liver transaminases. Serum bile acids greater than 10 umol/L in pregnancy are diagnostic of ICP. Elevated transaminases may also be seen but are not necessary for diagnosis.

Jaundice is estimated to affect 3–5 % of pregnancies worldwide, with the most common cause being viral hepatitis [5]. However, jaundice can also be seen in 10–25 % of ICP cases, the second leading cause [6]. Other causes include hyperemesis gravidarum, acute fatty liver of pregnancy, and pre-eclampsia. Beyond history and physical examination, evaluation of jaundice in pregnancy should include assessment of complete blood counts, hemolytic markers, bilirubin, and liver transaminases.

With respect to early-onset ICP, the differential diagnosis consists of extrahepatic and intrahepatic etiologies. Extrahepatic causes include choledocholithiasis, bile duct strictures, primary or secondary sclerosing cholangitis, and malignancy. Extrahepatic causes can be identified by ultrasound, MRCP, or CT. Intrahepatic causes include viral hepatitis, alcoholic hepatitis, adverse drug reactions, genetic mutations, and sepsis. Intrahepatic causes can be identified with history, physical exam, and laboratory studies.

As demonstrated in the present case, genetic variants can increase susceptibility to ICP. Aydin et al. 2020 obtained genetic testing for 25 patients with ICP and found alterations in the *ATP8B1*, *ABCB11*, and *ABCB4* genes [7]. The present patient's genetic variant in the ABCB11 gene, which is found on chromosome 2, encodes bile salt export pumps. Disruption of this gene leads to inhibition of bile acid transport to the bile duct [8]. Variants in this gene have been associated with the autosomal recessive genetic disorders mentioned previously, PFIC-2 and BRIC. These conditions vary in terms of phenotype. PFIC-2 is progressive and eventually leads to end-stage liver disease, while BRIC is characterized by self-limiting attacks of intense pruritis and jaundice interspersed with asymptomatic periods [9]. The present patient was suspected to have BRIC due to the remitting nature of her course and lack of liver dysfunction on evaluation. The hallmark of BRIC's pathophysiology is the accumulation of bile salts in bile canaliculi; however, the exact mechanism of cholestasis is poorly understood [10]. Although known triggers of BRIC include infection, contraceptive use, and pregnancy, literature addressing BRIC in pregnancy is limited and there are currently no guidelines for antepartum management [11].

Regardless of cause, management of ICP includes offering antenatal fetal surveillance at the time of diagnosis or a gestational age when delivery is both possible and desired following patient-centered counseling weighing the risks, benefits, and alternative treatments in relation to morbidity from ICP and prematurity. Patients with early-onset ICP should be monitored closely throughout the pregnancy and have bile acids and liver function testing every 1 to 2 weeks. As in the reported case, first-line treatment for ICP is ursodeoxycholic acid, though rifampin can be added for refractory cases. Delivery timing is based on serum bile acids. Currently, if bile acids are below 100 umol/L, delivery is recommended between 36 0/7 and 39 0/6 weeks of gestation [2]. If bile acids are above 100 umol/L, especially if transaminases are elevated, delivery is recommended by 36 0/7 weeks or at the time of diagnosis, given an increased risk of stillbirth [2]. In the setting of early-onset ICP with severely elevated bile acids, delivery even earlier than 36 weeks of gestation may also be considered, to decrease fetal risks.
